# Stripe-PZT Sensor-Based Baseline-Free Crack Diagnosis in a Structure with a Welded Stiffener

**DOI:** 10.3390/s16091511

**Published:** 2016-09-16

**Authors:** Yun-Kyu An, Zhiqi Shen, Zhishen Wu

**Affiliations:** 1 International Institute for Urban Systems Engineering, Southeast University, Nanjing 210096, China; 220131048@seu.edu.cn (Z.S.); zswu@seu.edu.cn (Z.W.); 2 Department of Architectural Engineering, Sejong University, Seoul 05006, Korea

**Keywords:** stripe-PZT sensor, Lamb wave, baseline-free crack diagnosis, welded stiffener, structural health monitoring, nondestructive testing

## Abstract

This paper proposes a stripe-PZT sensor-based baseline-free crack diagnosis technique in the heat affected zone (HAZ) of a structure with a welded stiffener. The proposed technique enables one to identify and localize a crack in the HAZ using only current data measured using a stripe-PZT sensor. The use of the stripe-PZT sensor makes it possible to significantly improve the applicability to real structures and minimize man-made errors associated with the installation process by embedding multiple piezoelectric sensors onto a printed circuit board. Moreover, a new frequency-wavenumber analysis-based baseline-free crack diagnosis algorithm minimizes false alarms caused by environmental variations by avoiding simple comparison with the baseline data accumulated from the pristine condition of a target structure. The proposed technique is numerically as well as experimentally validated using a plate-like structure with a welded stiffener, reveling that it successfully identifies and localizes a crack in HAZ.

## 1. Introduction

**Figure 1:**
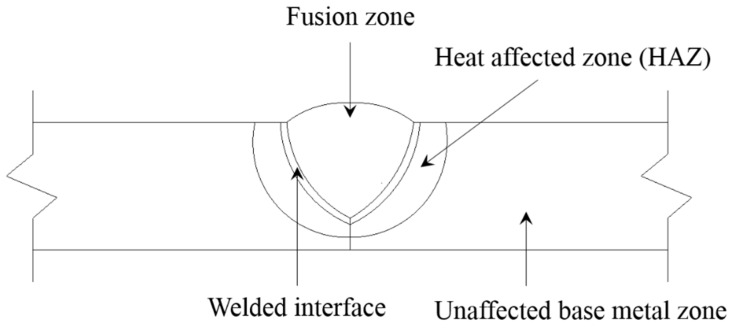
Cross section of a typical welded joint.

Structural health monitoring (SHM) has received much attention in recent years due to past catastrophic incidents involving civil infrastructures, which led to severe economic losses and casualties. To prevent and predict such catastrophic failures of civil infrastructures, local SHM techniques for early detection of incipient damage have been widely studied [1,2,3,4]. Although a number of local SHM techniques have been proposed, their application to in-situ civil infrastructures is still challenging due to the structural boundary complexity and harsh environmental conditions. In particular, welded area monitoring is important, but difficult to realize. Because the welding process-caused heat affected zone (HAZ) of the welded area is one of the structurally weakest areas [4,5], incipient cracks are often initiated from the HAZ due to excessive stress concentration even under yield stresses. After the heating process up to the melting temperature of the welding metal, shrinkage phenomena typically occur during the cooling process. Such shrinkage phenomena cause distortions in the longitudinal and circumferential directions and eventually lead to residual stresses due to the constraint boundary conditions, as shown in Figure 1. The fusion zone represents the welded area obtained with a welding rod. Between the fusion zone and HAZ, a welded interface exists. The other area where is not affected by the welding process is called the unaffected base metal zone. Once the structure is exposed to external loads, the residual stresses become one of the critical contributors to crack initiation in HAZ.

In this context, many researchers have tried to monitor cracks in HAZ. Sargent and Grondel et al., used Lamb waves generated and measured by lead zirconate titanate (PZT) to detect cracks in HAZ [6,7]. Then, Arone et al., tried to characterize HAZ defects through a non-contact ultrasonic technique [8]. Carvalho et al., utilized a magnetic flux leakage method to detect weld defects in pipes [9]. However, those techniques are baseline-dependent, meaning that a comparison process between current and baseline data is required to make a crack identification decision. Such a simple pattern comparison process of the Lamb wave signals may produce false alarms, because significant signal changes can be caused by not only a crack, but also operational and environmental variations [10].

To overcome this technical limitation, baseline-free crack diagnosis techniques that can identify cracks without comparing the currently measured data with the baseline data obtained from the pristine condition of the target structure have been proposed. An et al., developed a crack-induced mode conversion extraction technique for crack detection in HAZ and applied it to in-situ bridge monitoring [4]. Although the feasibility of the baseline-free techniques for real bridge monitoring was examined, they still have some technical limitations. Because the performance of the baseline-free techniques is highly dependent on the sensor installation conditions such as sensor size, location, bonding and wiring conditions, the sensor installation process must be expertly performed. Moreover, the sensor installation and cabling can be costly and labor-intensive, especially as the number of required sensors increases, which may cause more man-made errors resulting from implementation issues. Furthermore, cracks cannot be localized but only identified through the technique. More recently, non-contact laser ultrasonic scanning techniques have been developed to achieve baseline-free crack localization by measuring multi-spatial responses [11,12,13,14,15]. However, these techniques also have technical limitations in that: (1) laser scanning techniques can only be used for accessible or exposed surfaces of a target structure; (2) real-time monitoring is difficult to achieve through temporary laser scanning; and (3) the equipment required for precise laser ultrasonic scanning are relatively expensive.

To tackle the aforementioned technical issues, a new stripe-PZT sensor system and the corresponding baseline-free crack diagnosis algorithm are proposed in this study. The proposed technique has the following advantages: first, the applicability to in-situ civil infrastructures is significantly improved by delicately manufacturing embedded sensors with printed circuit wires and multi-channel connectors, making installation fast and convenient and minimizing man-made implementation errors. Moreover, baseline-free crack identification as well as localization can be accomplished using current multi-channel data simultaneously measured by a single stripe-PZT sensor system. Finally, real-time monitoring can be effectively achieved through the relatively cheap sensing system embedded into the target structure.

This paper is organized as follows: Section 2 introduces the theoretical background of Lamb wave interaction with a crack in HAZ and develops a novel baseline-free algorithm. Then, a finite element analysis is presented in Section 3. Subsequently, the experimental validation is shown in Section 4. Finally, this paper is concluded with an executive summary and brief discussion in Section 5.

## 2. Theoretical Development

### 2.1. Lamb Wave Interaction with a Crack in HAZ

**Figure 2:**
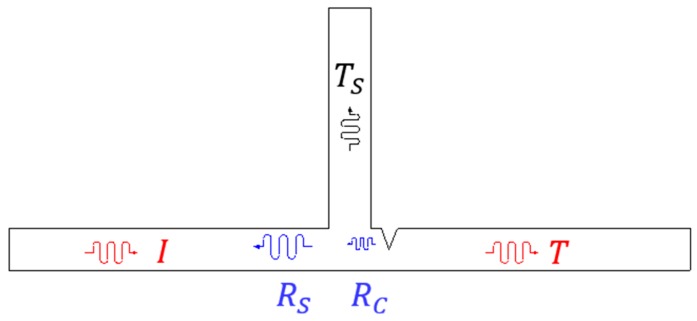
Schematic of Lamb wave propagation along a stiffened plate with a crack: *I*, *R_S_* and *R_C_* represent the incident wave, stiffener reflected wave and crack reflected wave, respectively. *T_S_* and *T* are the leaked wave to the stiffener and transmitted wave through the stiffener and crack, respectively.

Lamb waves have been widely used for crack detection in thin elastic structures, because they are sensitive to even incipient cracks and capable of traveling a long distance with little attenuation [16]. When Lamb waves propagating along a structure encounter a HAZ crack, complex scattering processes such as reflection, refraction, transmission and mode conversion occur. Figure 2 shows the typical Lamb wave propagation scheme on a plate-like structure with a welded stiffener used as the target structure in this study. If incident waves (*I*) propagating along the plate encounter the welded stiffener and the crack, a portion of waves are reflected from the stiffener (*R_S_*) as well as the crack (*R_C_*). Then, another wave portion is leaked to the vertical stiffener (*T_S_*), and others are transmitted through the crack (*T*). Although the actual wave interaction with HAZ may much more complicated, the principal wave components are only described in Figure 2. Such complicate physical interactions enable Lamb waves to characterize the crack. Among them, *R_C_* would be one of the most promising features for crack identification and localization if it can be extracted from the measured data. Note that all wave components might be mixed in the measured data. In the subsequent subsection, it is explained how *R_C_* can be isolated from the measured data.

### 2.2. Development of a Baseline-Free Crack Diagnosis Algorithm

This section develops the baseline-free crack diagnosis algorithm based on a frequency-wavenumber (*f-k*) domain analysis so that *R_C_* is extracted from the measured data. The *f-k* domain analysis has been recently used to differentiate ultrasonic wavefields according to their propagation directions in a specific frequency range of interest [11,12,17,18]. Thus, it is useful to analyze the complex wave scattering process caused by the wave interaction with a crack in HAZ.

**Figure 3:**
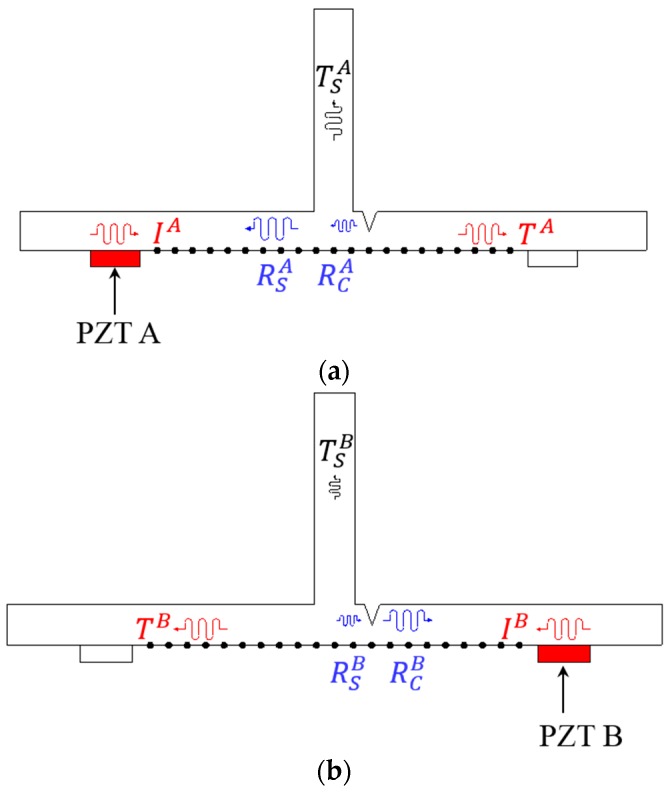
Schematics of Lamb wave generation and measurement: (**a**) $W_{T}^{A}$ generated from PZT A and (**b**) $W_{T}^{B}$ generated from PZT B. Superscripts A and B represent Lamb wave excited from PZT A and PZT B, respectively.

In order to use the *f-k* domain analysis, multi-spatial measurement data are required. In this study, spatially distributed multiple measurements are achieved through the two different Lamb wave excitation schemes as shown in Figure 3. First, Lamb waves are generated from PZT A, and the corresponding wavefields are measured at multiple spatial nodes, defined as $W_{T}^{A}$, as shown in Figure 3a. Similarly, Lamb waves are excited from PZT B, and their wavefields are acquired at the same spatial measurement nodes, coined as $W_{T}^{B}$, as shown in Figure 3b. The basic premise is that $W_{T}^{A}$ and $W_{T}^{B}$ should be measured at the same spatial nodes across the crack and stiffener locations, and the installation of two excitation PZTs, i.e., PZT A and PZT B should be identical and symmetric with respect to the stiffener. If there is no crack in Figure 3, $W_{T}^{A}$ and $W_{T}^{B}$ should be theoretically identical based on the dynamic reciprocal theorem [19]. However, if the asymmetric crack is initiated in HAZ as shown in Figure 3, the dynamic reciprocity will be broken. Based on such physical principle, the crack in HAZ can be identified and localized through the subsequent detailed procedure.

(1) Crack identification

Once $W_{T}^{A}$ and $W_{T}^{B}$ are measured in the time-space (*t-s*) domain, they are converted to the *f-k* domain using the 2D Fourier Transform [18]:

(1) $$U_{T}^{A}\left(k,\omega \right)=\overset{+\infty }{\underset{-\infty }{\iint }}W_{T}^{A}\left(x,t\right)e^{-i\left(kx+\omega t\right)}dxdt$$

(2) $$U_{T}^{B}\left(k,\omega \right)=\overset{+\infty }{\underset{-\infty }{\iint }}W_{T}^{B}\left(x,t\right)e^{-i\left(kx+\omega t\right)}dxdt$$

where $U_{T}^{A}$ and $U_{T}^{B}$ represent wavefields in the *f-k* domain converted from $W_{T}^{A}$ and $W_{T}^{B}$, respectively. *k*, *x*, *ω* and *t* denote the wavenumber, spatial coordinate, angular frequency and time, respectively.

Then, the backward wavefields of both PZT A and PZT B in the *f-k* domain, designated as $U_{B}^{A}$ and $U_{B}^{B}$, are computed by taking only positive *k* values from $U_{T}^{A}$ and $U_{T}^{B}$, respectively. Here, the sign of *k* physically means the wave propagation direction. The positive *k* values denote the backward wavefields ($U_{B}$) reflected from the stiffener or crack while the negative *k* values denote the forward wavefields ($U_{F}$). Subsequently, the cumulative energies of the backward wavefields in the *t-s* domain generated by PZT A and PZT B, defined as $E_{B}^{A}$ and $E_{B}^{B}$, respectively, are compared to determine the approximate crack location with respect to the vertical stiffener. To compute $E_{B}^{A}$ and $E_{B}^{B}$, $W_{B}^{A}$ and $W_{B}^{B}$ which represent backward wavefields generated by PZT A and PZT B in the *t-s* domain are calculated first:

(3) $$W_{B}^{A}\left(x,t\right)=\frac{1}{2\pi }\overset{+\infty }{\underset{-\infty }{\iint }}U_{B}^{A}\left(k,\omega \right)e^{-i\left(kx+\omega t\right)}dxdt$$

(4) $$W_{B}^{B}\left(x,t\right)=\frac{1}{2\pi }\overset{+\infty }{\underset{-\infty }{\iint }}U_{B}^{B}\left(k,\omega \right)e^{-i\left(kx+\omega t\right)}dxdt$$

Then, $E_{B}^{A}$ and $E_{B}^{B}$ can be computed as:

(5) $$E_{B}^{A}\left(x\right)=\int_{0}^{t}{\left[W_{B}^{A}\left(x,t\right)\right]}^{2}dt$$

(6) $$E_{B}^{B}\left(x\right)=\int_{0}^{t}{\left[W_{B}^{B}\left(x,t\right)\right]}^{2}dt$$

where $E_{B}^{A}$ and $E_{B}^{B}$ represent the energies of $W_{B}^{A}$ and $W_{B}^{B}$ cumulated up to a time point of *t*.

In Figure 3a, for example, $I^{A}$ encountering the stiffener will be firstly divided into $R_{S}^{A}$, $T_{S}^{A}$ and transmitted waves through the stiffener. And then, the transmitted waves will be separated into $R_{C}^{A}\;\mathrm{and}\;T^{A}$ due to the crack. Conversely, $R_{C}^{B}$ will be produced from $I^{B}$ first, and then the transmitted waves through the crack will be separated into $R_{S}^{B}$, $T_{S}^{B}$ and $T^{B}$ as shown in Figure 3b. Based on such physical phenomena, it is an obvious fact that $R_{C}^{B}$ is much larger than $R_{C}^{A}$ because Lamb waves are physically more reflected from the waveguide-decreased crack formation than the waveguide-increased vertical stiffener [20]. Moreover, $T_{S}^{A}$ is larger than $T_{S}^{B}$, physically meaning that more waves generated PZT A are leaked to the stiffener. Therefore, it can be concluded that $E_{B}^{B}$ is larger than $E_{B}^{A}$, when the crack is located near PZT B as depicted in Figure 3. Table 1 summarizes the crack identification criteria.

(2) Crack localization

After the crack is identified and roughly localized in the previous step, its precise localization can be achieved. First, the dynamic reciprocal difference ($\Delta U_{B}$) between $U_{B}^{A}$ and $U_{B}^{B}$ is computed as:

(7) $$\Delta U_{B}=\left|U_{B}^{A}-U_{B}^{B}\right|$$

Then, $\Delta R_{C}$ and $\Delta R_{S}$ are defined as:

(8) $$\Delta R_{C}=\left|R_{C}^{A}-R_{C}^{B}\right|$$

(9) $$\Delta R_{S}=\left|R_{S}^{A}-R_{S}^{B}\right|$$

Although physically $\Delta U_{B}$ contains both $\Delta R_{C}$ and $\Delta R_{S}$, $\Delta R_{C}$ is typically much larger than $\Delta R_{S}$ due to higher reflectivity of the crack than the stiffener. Based on the phenomenon, a threshold value (TR_1_) with respect to a one-sided 99% confidence interval is calculated to highlight $\Delta R_{C}$ and to minimize $\Delta R_{S}$ in $\Delta U_{B}$.

Subsequently, 2D Hanning window functions ($\Phi_{\omega }$ and $\Phi_{k}$) are employed with respect to $\Delta U_{B}$ in the *f* and *k* domains, respectively, so that the highlighted $\Delta R_{C}$ can be solely extracted from $\Delta U_{B}$ [20]:

(10) $$\Phi_{\omega }=\{\begin{array}{r} 0,\;\left|\omega -m_{\omega }\right|>2d_{\omega }\\ 0.5+0.5\cos\left[\frac{\pi \left\{\omega -m_{\omega }\right\}}{d_{\omega }}\right],\;\left|\omega -m_{\omega }\right|\leq 2d_{\omega } \end{array}\;\mathrm{for}\;k>0$$

(11) $$\Phi_{k}=\{\begin{array}{r} 0,\;\left|k-m_{k}\right|>2d_{k}\\ 0.5+0.5\cos\left[\frac{\pi \left\{k-m_{k}\right\}}{d_{k}}\right],\;\left|k-m_{k}\right|\leq 2d_{k} \end{array}\;\forall \omega$$

where $m_{\omega }$ and $2d_{\omega }$ denote the center and the width of $\Phi_{\omega }$ at a given ω, respectively. $m_{k}$ and $2d_{k}$ are the center and the width of $\Phi_{k}$ at a given k, respectively.

Once the $\Delta U_{B}$ values are projected on the *f* and *k* domains, the maximum and minimum ω and k values covering the projected $\Delta U_{B}$ values are computed. Then, the $m_{\omega }$ and $2d_{\omega }$ are determined as the mean value between the maximum and minimum ω values and the difference between the maximum and minimum ω values, respectively. Similarly, $m_{k}$ and $2d_{k}$ are determined as the mean value between the maximum and minimum *k* values and the difference between the maximum and minimum *k* values, respectively.

Then, the filtered wavefield in the *f-k* domain ($U_{Filter})$ is obtained using the following equation:

(12) $$U_{Filter}\left(k,\;\omega \right)=U_{T}\left(k,\;\omega \right)\cdot \Phi_{\omega }\left(k,\;\omega \right)\cdot \Phi_{k}\left(k,\;\omega \right)$$

where:

$$U_{T}=\{\begin{array}{c} U_{T}^{A},\;E_{B}^{A}>E_{B}^{B}\\ U_{T}^{B},\;E_{B}^{A}<E_{B}^{B} \end{array}$$

Subsequently, the filtered wavefield in the *t-s* domain ($W_{Filter})$ is reconstructed from $U_{Filter}$ using the 2D Inverse Fourier Transform:

(13) $$W_{Filter}\left(x,t\right)=\frac{1}{2\pi }\overset{+\infty }{\underset{-\infty }{\iint }}U_{Filter}\left(k,\omega \right)e^{-i\left(kx+\omega t\right)}dxdt$$

Then, $W_{F}$ and $W_{Filter}$ are converted to the frequency-time (*f-s*) domain using 1D Fourier Transform:

(14) $$W_{F,\omega \;}\left(x,\omega \right)=\overset{+\infty }{\underset{-\infty }{\int }}W_{F}\left(x,t\right)e^{-i\omega t}dt$$

(15) $$W_{Filter,\omega }\left(x,\omega \right)=\overset{+\infty }{\underset{-\infty }{\int }}W_{Filter}\left(x,t\right)e^{-i\omega t}dt$$

where $W_{F}$ represents the forward wavefields in the *t-s* domain converted from $U_{F}$. Once $W_{F,\omega \;}$ and $W_{Filter,\omega }$ are obtained, the crack can be precisely localized by computing the zero lag cross-correlation (ZLCC) between $W_{F,\omega \;}$ and $W_{Filter,\omega }$ in the *f-s* domain [21]:

(16) $$I\left(x\right)=\underset{\omega }{\sum }W_{F,\omega \;}\left(x,\omega \right)W_{Filter,\omega }^{*}(x,\omega )$$

where I(x) is the ZLCC value at a spatial node x. The superscript ‘∗’ is the complex conjugate. Note that the ZLCC computation in the frequency domain is more effective than the time domain computation in terms of saving the computational costs.

When ZLCC physically representing the extracted *R_C_* encounters $W_{F}$ coming from *I* as shown in Figure 2, their interaction momentarily generates standing wave components in the vicinity of the crack [18]. Here, the standing waves are produced when *R_C_* and *I* have the identical wavelength and frequency conditions. Such standing wave phenomenon satisfies the ZLCC condition physically meaning that the similarity indicator of two data series having zero-delayed or in-phase. Therefore, the ZLCC values abruptly increase where crack-induced standing waves are generated compared to the intact region.

After the ZLCC values are computed at all spatial nodes of interest, a threshold value (TR_2_) with respect to a one-sided 99% confidence interval is calculated to minimize false alarms. Even though ZLCC is the effective crack indicator, the computed ZLCC values may have noise components. Finally, the precise crack location is highlighted where the ZLCC value exceeds TR_2_.

## 3. Finite Element (FE) Analysis

### 3.1. Description of a FE Model

**Figure 4:**
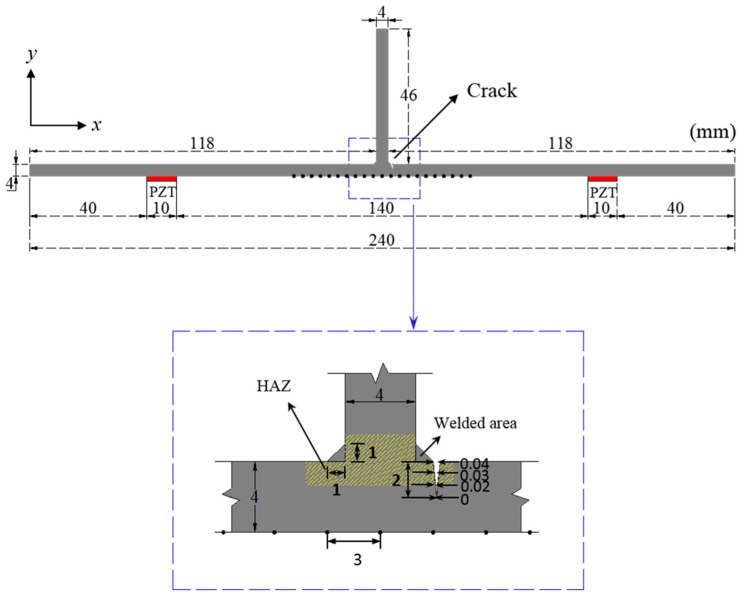
2D plane strain model with a vertical stiffener: The PZTs with a dimension of 10 × 0.508 mm^2^ are modeled on the opposite surface to the vertical stiffener for Lamb wave generation. The depth of the crack is 2 mm and its width is varying from 0 to 40 μm along the through-the-thickness direction at HAZ.

To validate the proposed technique, a 2D plane strain FE model is made using ABAQUS/Standard 6.13 [22]. As shown in Figure 4, the PZTs are modeled on the opposite surface to the vertical stiffener. They are APC 850 type [23] with dimensions of 10 × 0.508 mm^2^. The crack depth is 2 mm, and its width varies from 0 to 40 μm along the through-the-thickness direction. In particular, the crack is introduced at HAZ as shown in Figure 4. The material properties of the FE model are summarized in Table 2.

The PZTs attached on the surface are used to generate Lamb waves by applying the input waveform of 7-cycle toneburst signals with the driving frequencies of 100 kHz and 150 kHz. The mesh size of 1 × 1 mm^2^ and the sampling rate of 20 MHz are determined by the spatial discretization rule [24]:

(17) $$\max\left(\Delta x,\;\Delta y\right)<\delta_{\min}/10,\Delta t<0.7\min\left(\Delta x,\;\Delta y\right)/C_{L}$$

where Δx, Δy, $\delta_{\min}$ and $C_{L}$ represent the mesh size in *x* direction, *y* direction, minimum wave length and longitudinal wave velocity, respectively.

To ensure the performance of the *f-k* domain analysis, sensing nodes should contain at least a single wavelength of Lamb wave mode. More than 21 discrete sensing nodes with an identical spatial interval of 3 mm are required in this model because the longest wavelengths of fundamental symmetric (S_0_) and antisymmetric (A_0_) modes are about 62.91 mm and 31.7 mm when the driving frequency is 100 kHz. Note that the wavelengths of S_0_ and A_0_ modes at 150 kHz are 41.94 mm and 21.13 mm, respectively.

### 3.2. FE Analysis Results

#### 3.2.1. Crack Identification

**Figure 5:**
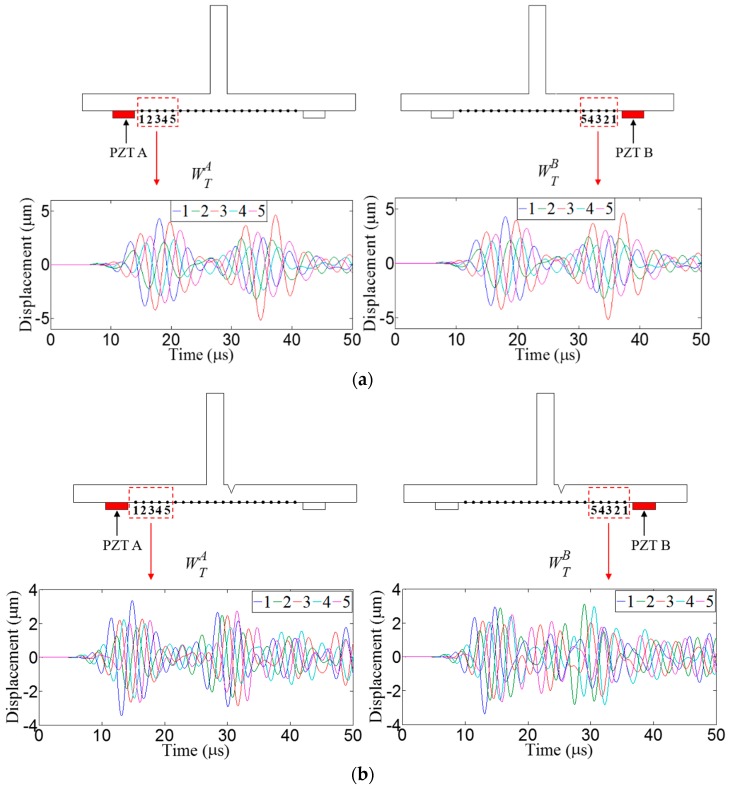
Representative $W_{T}^{A}$ and $W_{T}^{B}$ obtained from (**a**) intact and (**b**) crack models when the driving frequency is 150 kHz.

Figure 5 shows the representative $W_{T}^{A}$ and $W_{T}^{B}$ obtained from the intact and crack models at 150 kHz. As expected, $W_{T}^{A}$ and $W_{T}^{B}$ are exactly same in the intact case of Figure 5a, meaning that the dynamic reciprocity is retained. On the other hand, the crack-caused signal difference between $W_{T}^{A}$ and $W_{T}^{B}$ is clearly observed in Figure 5b.

**Figure 6:**
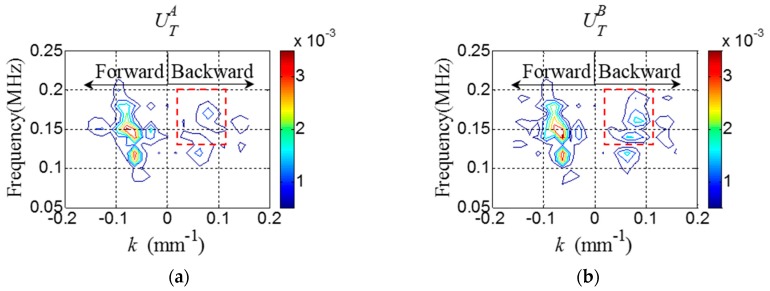
*f-k* domain plots (**a**) $U_{T}^{A}$ and (**b**) $U_{T}^{B}$ obtained from the crack model at 150 kHz.

Once $W_{T}^{A}$ and $W_{T}^{B}$ are obtained at all spatial sensing nodes of interest, the *f-k* domain plots can be obtained using Equations (1) and (2). Figure 6 shows the representative *f-k* domain plots of the crack model at 150 kHz. The crack-induced difference between $U_{T}^{A}$ and $U_{T}^{B}$ in the backward wavefiled area of the *f-k* domain plots is more clearly observed in Figure 6. From the *f-k* domain data, $E_{B}^{A}$ and $E_{B}^{B}$ are computed as 1.2 × 10^−15^ and 1.37 × 10^−15^, respectively, indicating that crack location is the PZT B side with respect to the stiffener. This diagnosis result shows the good agreement with the actual crack location as shown in Figure 5b.

#### 3.2.2. Crack Localization

**Figure 7:**
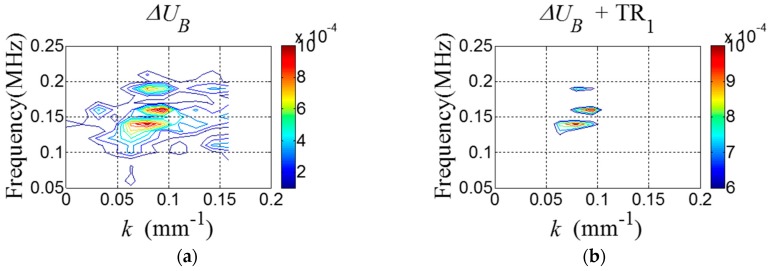
*f-k* domain plots of (**a**) $\Delta U_{B}$ and (**b**) $\Delta U_{B}$ after applying TR_1_.

Figure 7a shows $\Delta U_{B}$ containing $\Delta R_{C}$ and $\Delta R_{S}$. By applying TR_1_ to $\Delta U_{B}$, $\Delta R_{C}$ is highlighted in Figure 7b. Then, the 2D Hanning window parameters are computed using Equations (10) and (11). Here, $d_{\omega }=0.0232$, $m_{\omega }=0.08214$, $d_{k}=0.0841$ and $m_{k}=0.1636$.

**Figure 8:**
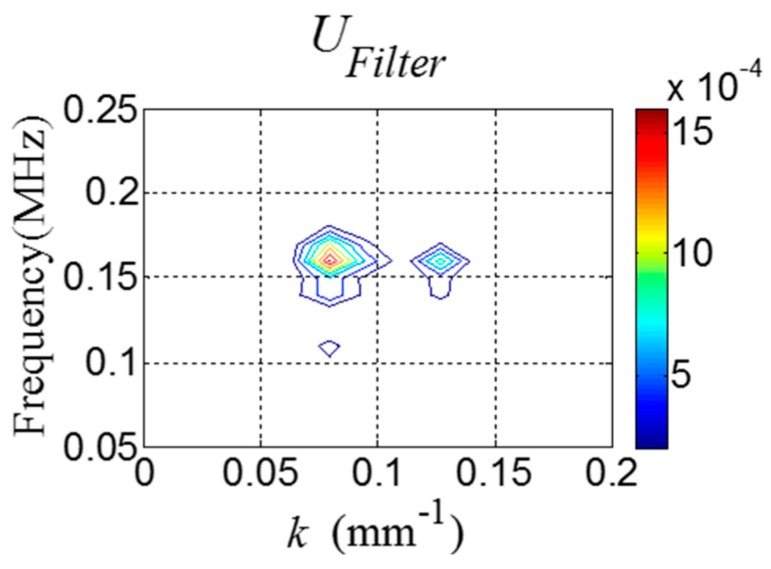
*f-k* domain plot of $U_{Filter}$.

Next, $U_{Filter}$ is obtained using Equation (12) as shown in Figure 8.

**Figure 9:**
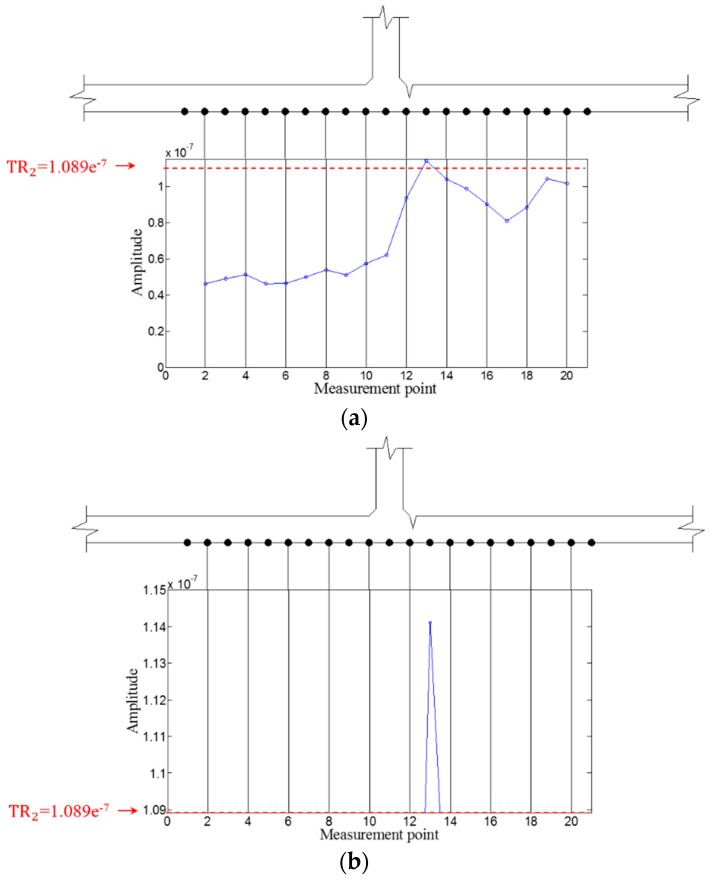
ZLCC values of each measurement node in the crack case at 100 kHz (**a**) before and (**b**) after applying TR_2_.

**Figure 10:**
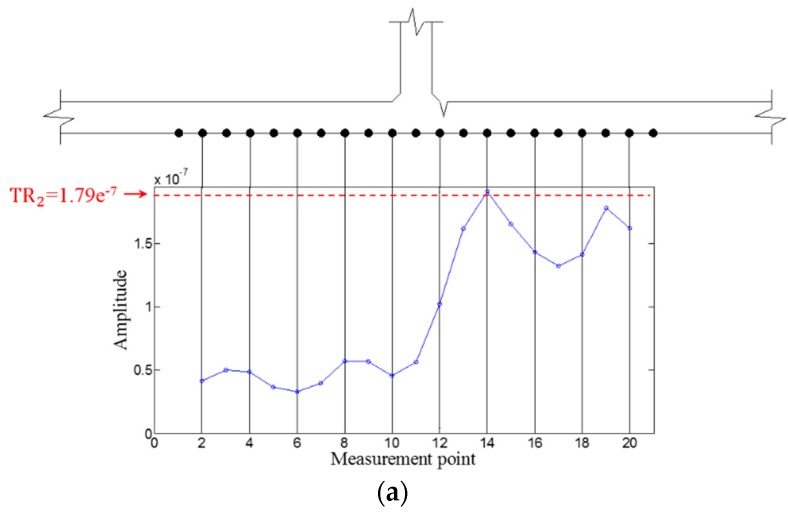

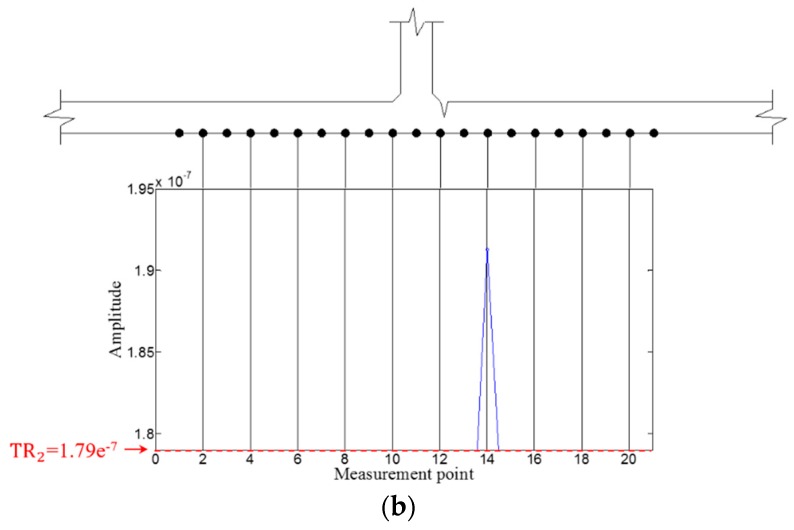
ZLCC values of each measurement node in the crack case at 150 kHz (**a**) before and (**b**) after applying TR_2_.

Then, $W_{Filter}$ is computed using Equation (13), and $W_{F,\omega }$ and $W_{Filter,\omega }$ are subsequently obtained using Equations (14) and (15) for the ZLCC calculation. Using Equation (16), the ZLCC values of each sensing node are obtained as shown in Figure 9 and Figure 10. Figure 9a shows the ZLCC values at 100 kHz, and the ZLCC values exceeding TR_2_ indicate the previse crack location as shown in Figure 9b. The crack location indicated as the sensing node #13, which has 3 mm error compared to the actual one as shown in Figure 9b. Similarly, the 150 kHz case reveals the larger error of 6 mm than the 100 kHz case as displayed in Figure 10. This localization errors may come from the standing wave generation mechanism which are physically produced in front of the crack when it comes to the excitation PZT side. Then, it can be seen that the localization error depends on the driving wavelength. Although the results show some localization errors, it can be acceptable by considering the fact that the minimum sensing spatial interval is 3 mm.

## 4. Experimental Validation

### 4.1. Development of a Stripe-PZT Sensor

**Figure 11:**
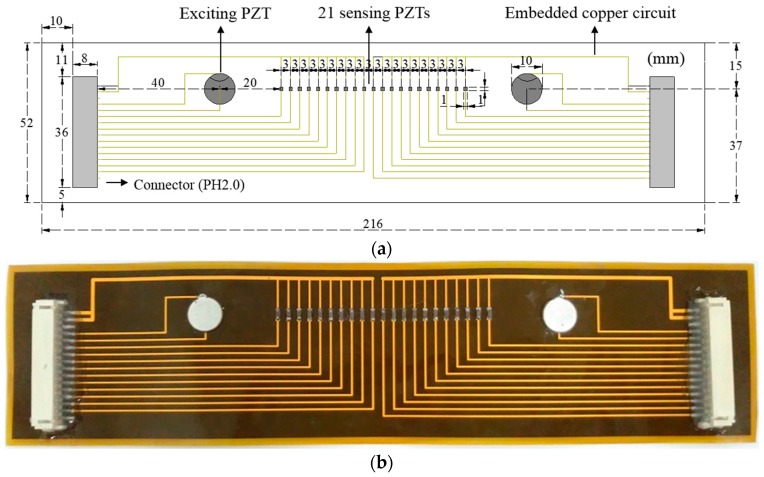
Stripe-PZT sensor design: (**a**) schematic design of the stripe-PZT sensor (**b**) actual stripe-PZT sensor manufactured with a flexible printed circuit board (PCB).

To experimentally validate the proposed technique, the stripe-PZT sensor is developed as shown in Figure 11. The stripe-PZT sensor enables to simultaneously generate Lamb waves and measure $W_{T}^{A}$ and $W_{T}^{B}$ without additional sensor installation. Figure 11a shows that the stripe-PZT sensor consists of the two circular excitation PZTs, 21 sensing PZTs with the spatial interval of 3 mm, and two connectors (PH 2.0) for users’ convenience. All components are sophisticatedly embedded onto flexible printed circuit board (PCB) as shown in Figure 11 so that the stripe-PZT sensor can be applied to curved surfaces. The actual stripe-PZT sensor is displayed in Figure 11b.

### 4.2. Description of Experimental Setup

**Figure 12:**
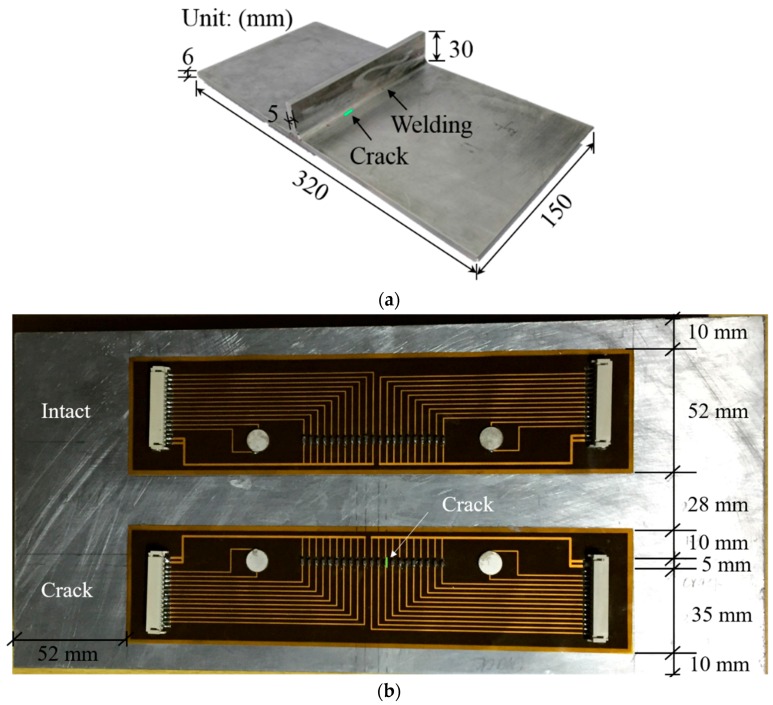
Target structure with the stripe-PZT sensors: (**a**) a vertically stiffened aluminum specimen with a crack and (**b**) installation of the stripe-PZT sensors on the opposite surface of the stiffener and the crack.

The target structure for experimental validation is an A6061 aluminum plate with a vertical stiffener as shown in Figure 12a. The vertically stiffened aluminum plate is manufactured by welding the stiffener to the plate. Then, an artificial notch with a dimension of 5 × 1 × 3 mm^3^ is introduced at HAZ as shown in Figure 12a. It has been reported that a notch can properly represent an open crack if the notch width is trivial compared to the smallest wavelength of the measured Lamb waves [25]. In particular, the crack is made at the HAZ area 50 mm apart from the end boundary along the stiffener because both intact and crack areas can be tested using the same specimen as shown in Figure 12b. Two stripe-PZT sensors are installed on the opposite surface of the crack and stiffener as displayed in Figure 12b. The upper one crosses only the welded stiffener, and the lower one covers both the crack and the stiffener.

**Figure 13:**
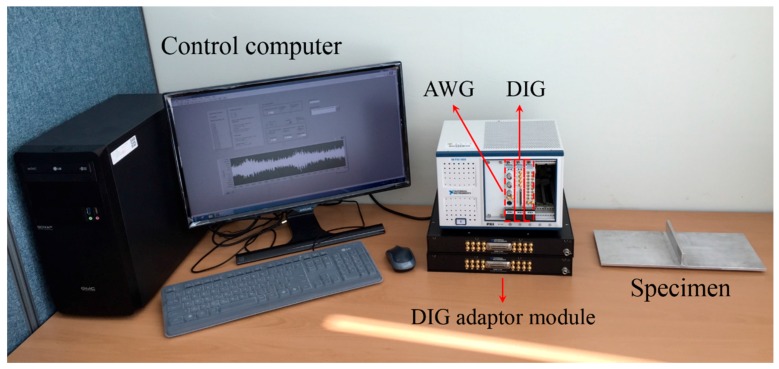
Experimental setup consisted of a control computer, an arbitrary waveform generator (AWG), a multi-channel digitizer (DIG), DIG adaptor modules and the specimen.

Figure 13 shows experimental setup consisted of a control computer, an arbitrary waveform generator (AWG), a digitizer (DIG) and DIG adaptor modules. The control computer sends out the control signal and 7-cycle toneburst input waveform to AWG. Then, AWG sends out the input waveform to the excitation PZTs of the stripe-PZT sensor shown in Figure 11 to generate Lamb waves. Meanwhile, the corresponding responses are simultaneously measured by the spatially distributed sensing PZTs at one time using the multi-channel DIG. Note that two 16 channel DIG adaptor modules are used to gather the responses. The measured data are transmitted to the control computer and stored for the automatic signal processing. In the tests, two different driving frequencies of 100 kHz and 150 kHz are used as the same condition as the FE simulation one, and the sampling rate of 10 MHz is used. 40 μs time signals are measured 100 times, averaged in the time domain, and bandpass-filtered with 10 kHz and 300 kHz cutoff frequencies to improve the signal-to-noise ratio.

### 4.3. Experimental Results

#### 4.3.1. Crack Identification

**Figure 14:**
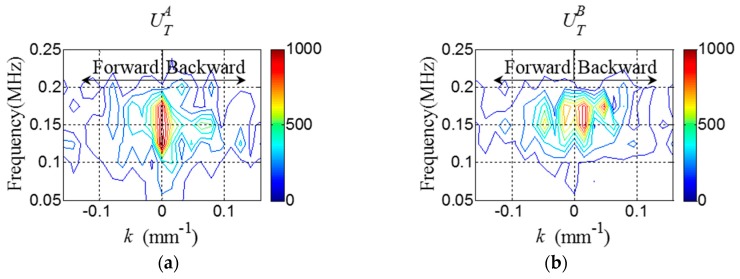
*f-k* domain plots (**a**) $U_{T}^{A}$ and (**b**) $U_{T}^{B}$ obtained from the cracked area of the specimen at 150 kHz.

**Figure 15:**
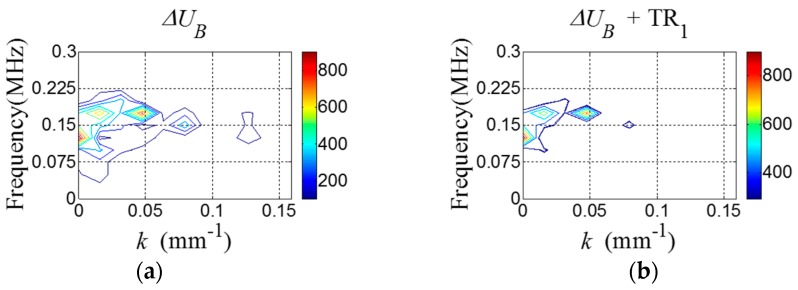
*f-k* domain plots of (**a**) $\Delta U_{B}$ and (**b**) $\Delta U_{B}$ corresponding to Figure 13 after applying TR_1_.

Once $W_{T}^{A}$ and $W_{T}^{B}$ are obtained from the target structure, the *f-k* domain analysis is subsequently carried out. Figure 14 shows the representative *f-k* domain plots of $W_{T}^{A}$ and $W_{T}^{B}$ obtained from the cracked area of the specimen at 150 kHz. The crack-induced difference between $U_{T}^{A}$ and $U_{T}^{B}$ can be observed in Figure 14, and $\Delta U_{B}$ caused by the crack is more clearly shown in Figure 15. Similarly, $E_{B}^{A}$ and $E_{B}^{B}$ are computed as 1.95 × 10^−4^ and 2.1 × 10^−4^, respectively, indicating that crack location is the PZT B side with respect to the stiffener.

#### 4.3.2. Crack Localization

Figure 15a shows $\Delta U_{B}$ containing $\Delta R_{C}$ and $\Delta R_{S}$. By applying TR_1_ to $\Delta U_{B}$, $\Delta R_{C}$ is highlighted in Figure 15b. Then, the 2D Hanning window parameters are computed using Equations (10) and (11). Here, $d_{\omega }=0.05625$, $m_{\omega }=0.15$, $d_{k}=0.0409$, $m_{k}=0.0409$.

**Figure 16:**
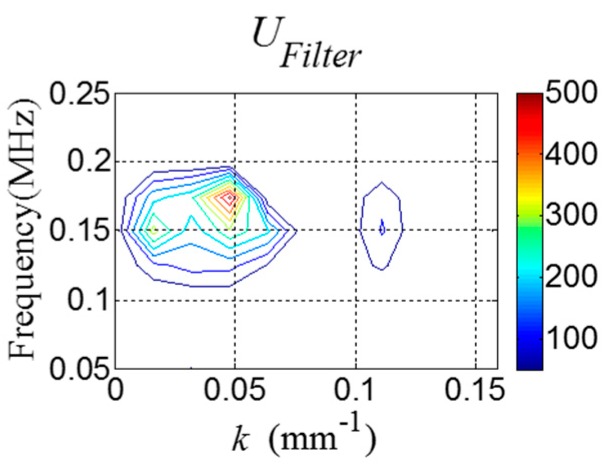
*f-k* domain plot of $U_{Filter}$ computed from Figure 14.

Next, $U_{Filter}$ is obtained using Equation (12) as shown in Figure 16.

**Figure 17:**
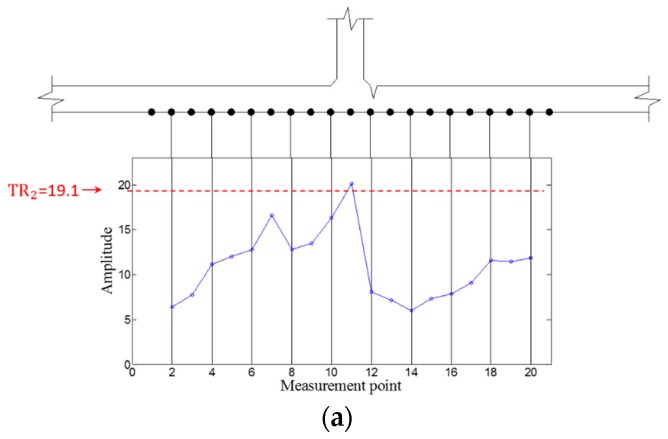

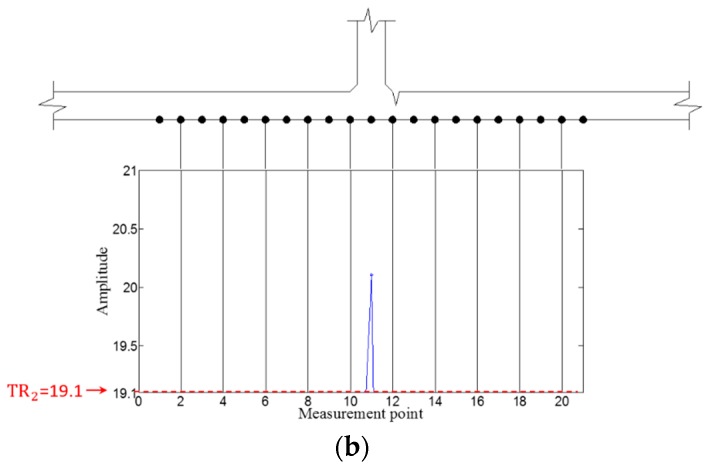
ZLCC values obtained from the crack area at 100 kHz (**a**) before and (**b**) after applying TR_2_.

**Figure 18:**
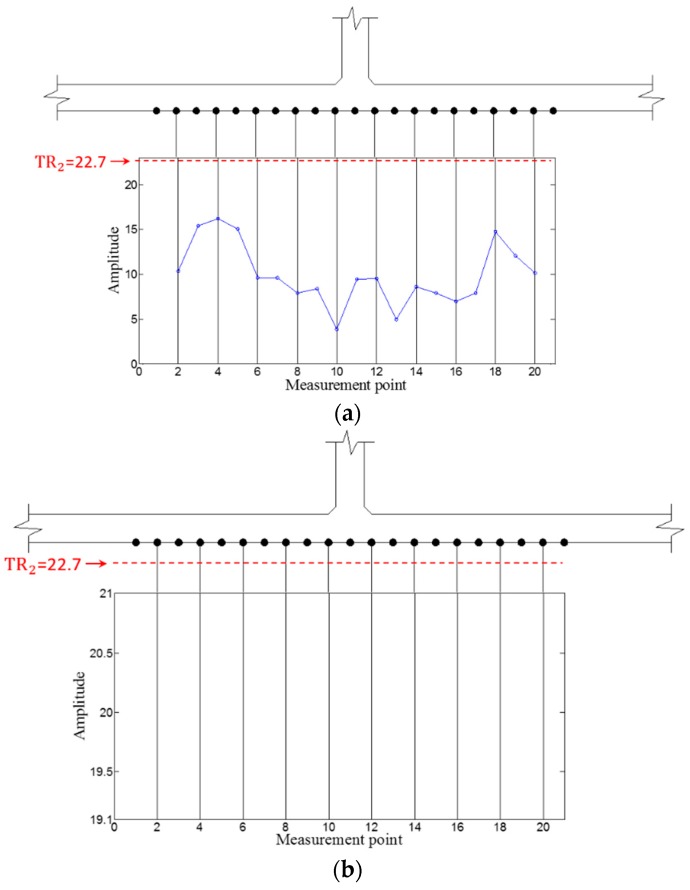
ZLCC values obtained from the intact area at 100 kHz (**a**) before and (**b**) after applying TR_2_.

**Figure 19:**
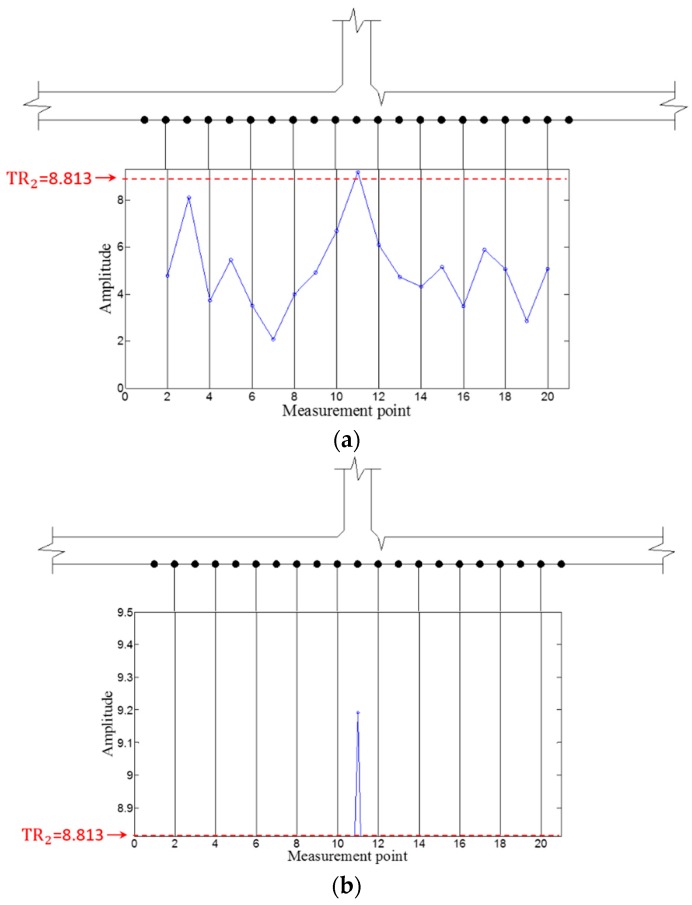
ZLCC values obtained from the crack area at 150 kHz (**a**) before and (**b**) after applying TR_2_.

**Figure 20:**
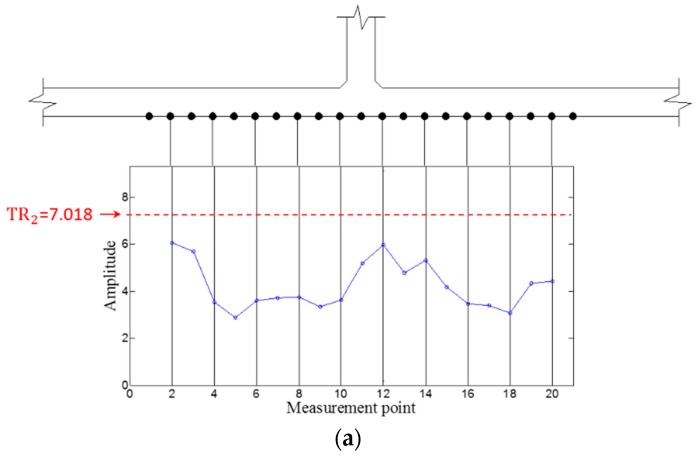

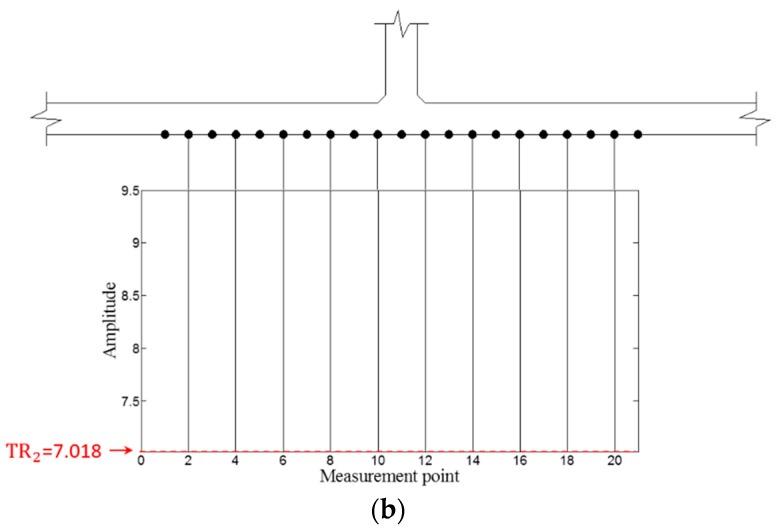
ZLCC values obtained from the intact area at 150 kHz (**a**) before and (**b**) after applying TR_2_.

Then, $W_{Filter}$ is computed using Equation (13), and $W_{F,\omega \;}$ and $W_{Filter,\omega }$ are subsequently obtained using Equations (14) and (15). Similarly, the ZLCC values of each sensing node in intact and cracked area are obtained using Equation (16) as displayed in Figure 17, Figure 18, Figure 19 and Figure 20. In both cases of 100 kHz and 150 kHz, the crack locations are estimated as the sensing point #11, which has 3 mm error compared to the actual one as shown in Figure 17b and Figure 19b. Due to the imperfection of the stripe-PZT sensor installation and measurement noises might be major error sources. Note that such noise sources can similarly affect to the intact case as well. Although the ZLCC values of the intact cases shown in Figure 18 and Figure 20 should be theoretically zero, there are some values below TR_2_ due to the noise sources. Nevertheless, no positive false alarm is indicated after applying TR_2_ in the intact cases as observed in Figure 18b and Figure 20b. Again, the most significant conclusions are that (1) there is no negative as well as positive false alarm in all tested cases; (2) the fatigue crack at HAZ is automatically localized without any baseline data and experts’ intervention; and (3) the sensor installation is much easier, and sensing time is much shorter than the existing ultrasonic nondestructive testing techniques, making it possible to minimize measurement error sources and enhance the applicability to real structures.

## 5. Conclusions

This paper has proposed a stripe-PZT sensor system and the corresponding baseline-free crack evaluation algorithm. Next they are numerically and experimentally validated, revealing that the proposed technique can successfully identify and localize fatigue cracks. The main achievements of the proposed technique lie in that: (1) It can significantly reduce man-made errors due to handling mistakes; (2) Since all sensors are embedded onto a printed circuit board in the stripe-PZT sensor system, its installation is much easier and faster; (3) Baseline-free crack identification and localization can be achieved using only currently measured data even at a welded stiffened area, making it less vulnerable to false alarms due to environmental and operational variations. However, there are some technical challenges to be overcome for in-situ applications. The stripe-PZT sensor should be installed symmetrically with respect to the welded stiffener. Closed type fatigue cracks may not be detected correctly. As follow-up studies, the performance with more complex boundary conditions is now being investigated. Furthermore, validation tests will be performed under various environmental conditions such as different temperature and external loading variations. Finally, the proposed technique will be applied to in-situ structures.

## Figures and Tables

**Table 1:** Crack identification criteria.

(1) $E_{B}^{A}>E_{B}^{B}$	Crack on the PZT A side
(2) $E_{B}^{A}<E_{B}^{B}$	Crack on the PZT B side
(3) $E_{B}^{A}=E_{B}^{B}$	No crack

**Table 2:** Material properties of the plate model: Mass density (**ρ**), longitudinal wave velocity (***C_L_***), shear wave velocity (***C_T_***), Young’s modulus (**E**) and Poisson coefficient (**υ**).

ρ (kg/m^3^)	*C_L_* (m/s)	*C_T_* (m/s)	E (GPa)	υ
2620.4	6291	3170	70	0.33
